# Supplementary material for: Molecular dynamics simulations of human cohesin subunits identify DNA binding sites and their potential roles in DNA loop extrusion
Source: PLoS Comput Biol. 2025 Apr 4;21(4):e1012493. doi: 10.1371/journal.pcbi.1012493 (PMC11970657; doi:10.1371/journal.pcbi.1012493)
Supplement: S1 Fig — (A) Sequences of Homo sapiens NIPBL, Saccharomyces cerevisiae SCC2, and Schizosaccharomyces pombe MIS4 protein aligned using the T-coffee server [38]. T-coffee combines popular MSA algorithms and evaluates the alignment confidence of each column by transitive consistency score (TCS). TCS indicates the consistency between different MSA algorithms. Therefore, a high score indicates better sequence conservation. Protein structures are indicated below TCS scores, with rectangles indicating structured domains and lines indicating intrinsically disordered regions. (B) Sequences of Homo sapiens STAG1, Saccharomyces cerevisiae SCC3, and Schizosaccharomyces pombe PSC3 protein aligned using the T-coffee server. (PDF) [file pcbi.1012493.s001.pdf]

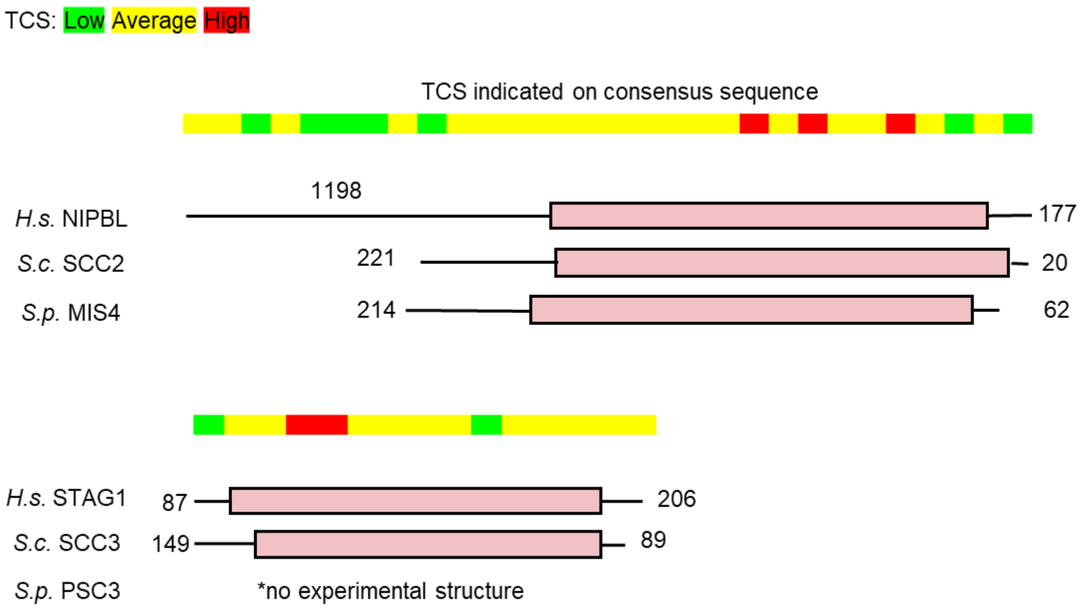

Fig S1. Sequence and structure alignment of HAWK subunits in human and yeast cohesin. (A) Sequences of *Homo sapiens* NIPBL, *Saccharomyces cerevisiae* SCC2, and *Schizosaccharomyces pombe* MIS4 protein aligned using the T-coffee server (1). T-coffee combines popular MSA algorithms and evaluates the alignment confidence of each column by transitive consistency score (TCS). TCS indicates the consistency between different MSA algorithms. Therefore, a high score indicates better sequence conservation. Protein structures are indicated below TCS scores, with rectangles indicating structured domains and lines indicating intrinsically disordered regions. (B) Sequences of *Homo sapiens* STAG1, *Saccharomyces cerevisiae* SCC3, and *Schizosaccharomyces pombe* PSC3 protein aligned using the T-coffee server.

1. Notredame C, Higgins DG, Heringa J. T-coffee: a novel method for fast and accurate multiple sequence alignment1. Journal of Molecular Biology. 2000 Sep 8;302(1):205–17
