# Supplementary material for: Molecular dynamics simulations of human cohesin subunits identify DNA binding sites and their potential roles in DNA loop extrusion
Source: PLoS Comput Biol. 2025 Apr 4;21(4):e1012493. doi: 10.1371/journal.pcbi.1012493 (PMC11970657; doi:10.1371/journal.pcbi.1012493)
Supplement: S2 Fig — (A) SMC1 head domain with emanating partial coiled coil arm. (B) SMC3 head domain with emanating partial coiled coil arm. (C) SMC1/3 head dimer with emanating partial coiled coil arms. (D) SMC1 hinge domain. (E) SMC3 hinge domain. (F) SMC1/3 hinge dimer. (G) the HEAT repeats domain of STAG1. (H) the HEAT repeats domain of NIPBL. (PDF) [file pcbi.1012493.s002.pdf]

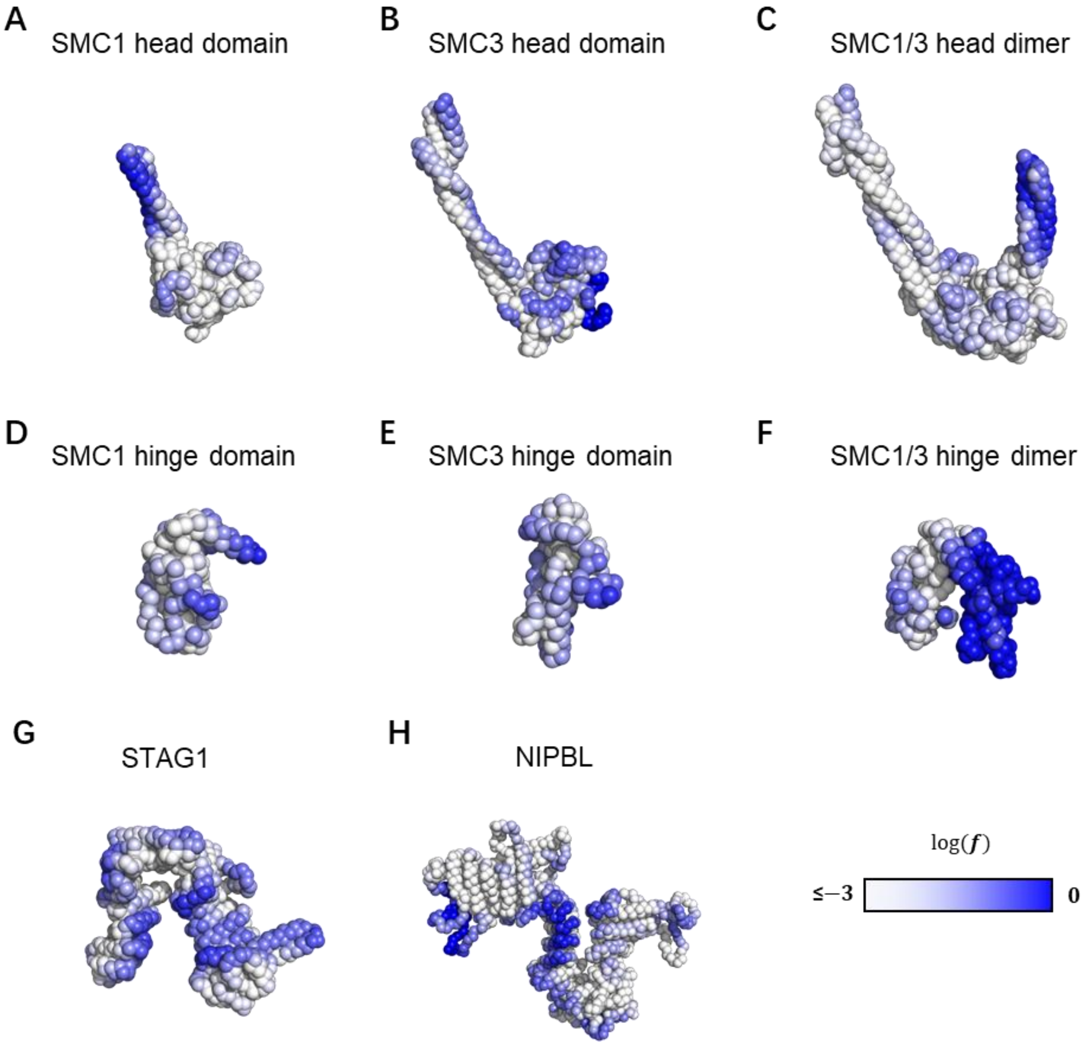

Fig S2. DNA contact frequency of each amino acid mapped on subunit structures. Contact frequency f of each amino acid residue particle is defined as $f = N/N_0$, where N is number of frames the corresponding particle is in contact with any DNA segment, N_0 is the total number of frames in all simulation trajectories. (A) SMC1 head domain with emanating partial coiled coil arm. (B) SMC3 head domain with emanating partial coiled coil arm. (C) SMC1/3 head dimer with emanating partial coiled coil arms. (D) SMC1 hinge domain. (E) SMC3 hinge domain. (F) SMC1/3 hinge dimer. (G) the HEAT repeats domain of STAG1. (H) the HEAT repeats domain of NIPBL.
