# Supplementary material for: Molecular dynamics simulations of human cohesin subunits identify DNA binding sites and their potential roles in DNA loop extrusion
Source: PLoS Comput Biol. 2025 Apr 4;21(4):e1012493. doi: 10.1371/journal.pcbi.1012493 (PMC11970657; doi:10.1371/journal.pcbi.1012493)
Supplement: S3 Fig — (A) definition of the DNA contact pattern similarity between two amino acid residues through Jaccard distance. (B) hierarchical clustering of surface amino acid residue particles of SMC1 head domain. (PDF) [file pcbi.1012493.s003.pdf]

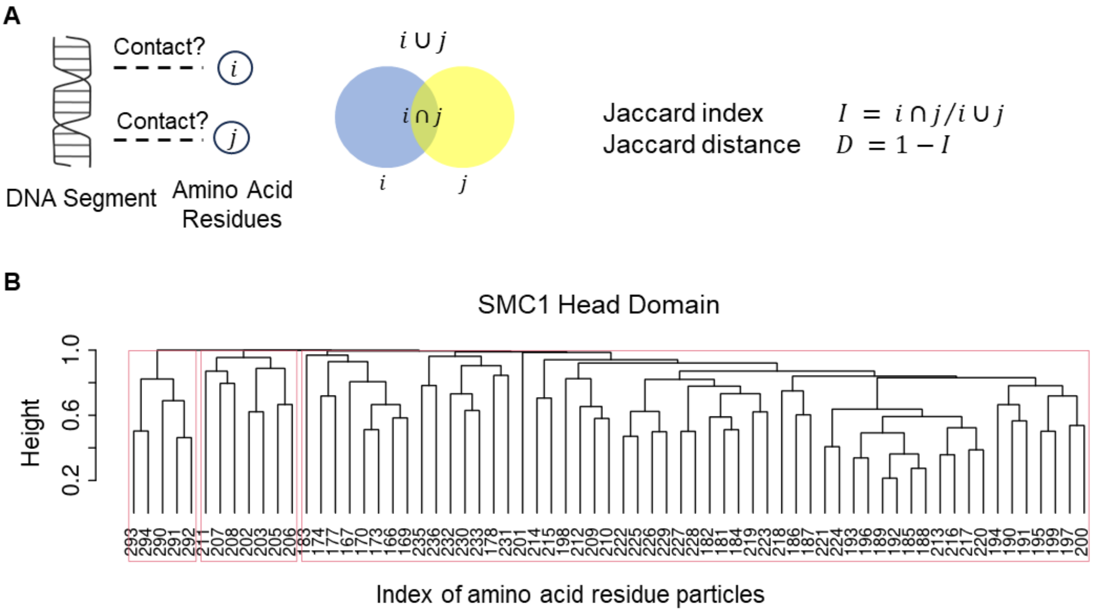

Fig S3. Illustration of DNA binding-patch calling method. (A) definition of the DNA contact pattern similarity between two amino acid residues through Jaccard distance. (B) hierarchical clustering of surface amino acid residue particles of SMC1 head domain.
