# Supplementary material for: Molecular dynamics simulations of human cohesin subunits identify DNA binding sites and their potential roles in DNA loop extrusion
Source: PLoS Comput Biol. 2025 Apr 4;21(4):e1012493. doi: 10.1371/journal.pcbi.1012493 (PMC11970657; doi:10.1371/journal.pcbi.1012493)
Supplement: S4 Fig — (A) SMC1 head domain with emanating partial coiled coil arm. (B) SMC3 head domain with emanating partial coiled coil arm. (C) SMC1/3 head dimer with emanating partial coiled coil arms. (D) SMC1 hinge domain. (E) SMC3 hinge domain. (F) SMC1/3 hinge dimer. (G) the HEAT repeats domain of STAG1. (H) the HEAT repeats domain of NIPBL. (PDF) [file pcbi.1012493.s004.pdf]

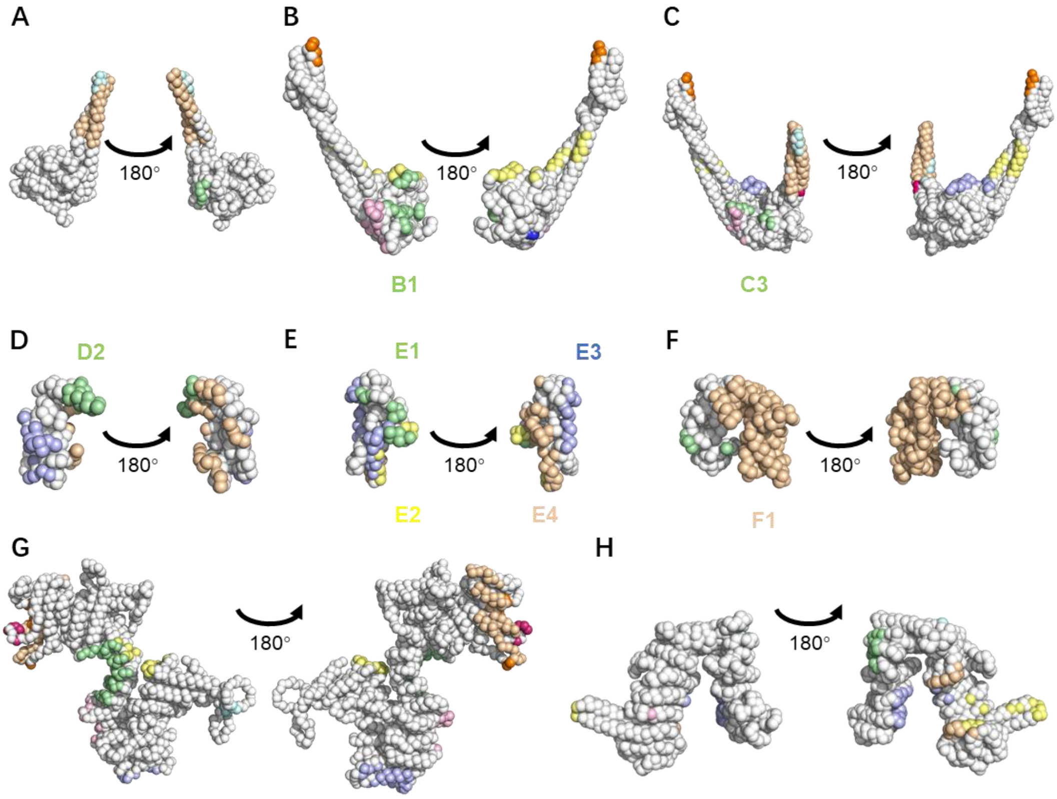

Fig S4. Identified DNA binding patches mapped on subunit structures. Patches affected by SMC1/SMC3 head dimerization and SMC1/SMC3 hinge dimerization are labeled in figures. (A) SMC1 head domain with emanating partial coiled coil arm. (B) SMC3 head domain with emanating partial coiled coil arm. (C**)** SMC1/3 head dimer with emanating partial coiled coil arms. **(**D) SMC1 hinge domain. (E) SMC3 hinge domain. (F) SMC1/3 hinge dimer. (G) the HEAT repeats domain of STAG1. (H) the HEAT repeats domain of NIPBL.
