# Supplementary material for: Molecular dynamics simulations of human cohesin subunits identify DNA binding sites and their potential roles in DNA loop extrusion
Source: PLoS Comput Biol. 2025 Apr 4;21(4):e1012493. doi: 10.1371/journal.pcbi.1012493 (PMC11970657; doi:10.1371/journal.pcbi.1012493)
Supplement: S5 Fig — The logarithm ratio of contact events with lifetime τ larger than time t was plotted as a function of t. The exponential decay phase with a smaller dissociation rate constant indicating binding events was plotted as green data points, and the linear models fitted using these data points were plotted as green lines. (PDF) [file pcbi.1012493.s005.pdf]

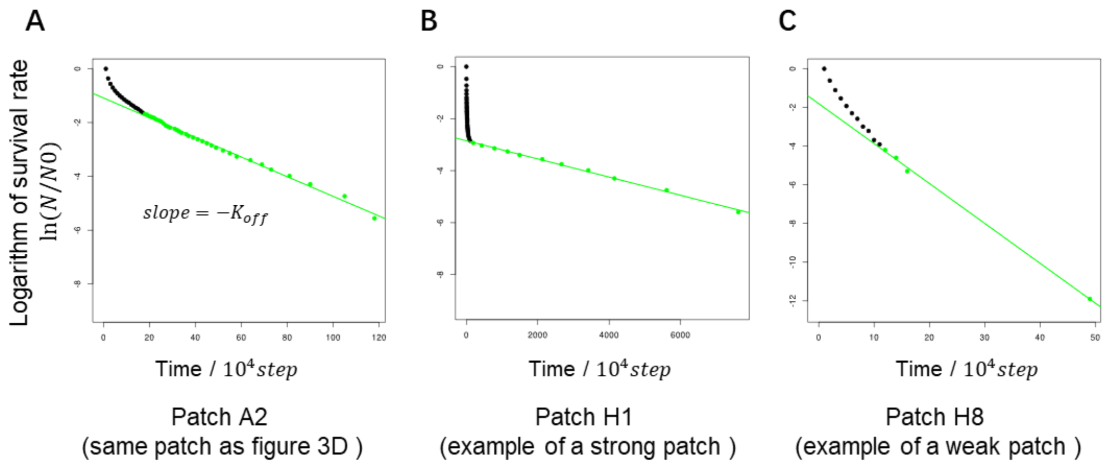

Fig S5. Survival rate–time distribution of DNA-patch contact events. The logarithm ratio of contact events with lifetime τ larger than time t was plotted as a function of t. The exponential decay phase with a smaller dissociation rate constant indicating binding events was plotted as green data points, and the linear models fitted using these data points were plotted as green lines.
