# Supplementary material for: Molecular dynamics simulations of human cohesin subunits identify DNA binding sites and their potential roles in DNA loop extrusion
Source: PLoS Comput Biol. 2025 Apr 4;21(4):e1012493. doi: 10.1371/journal.pcbi.1012493 (PMC11970657; doi:10.1371/journal.pcbi.1012493)
Supplement: S6 Fig — (PDF) [file pcbi.1012493.s006.pdf]

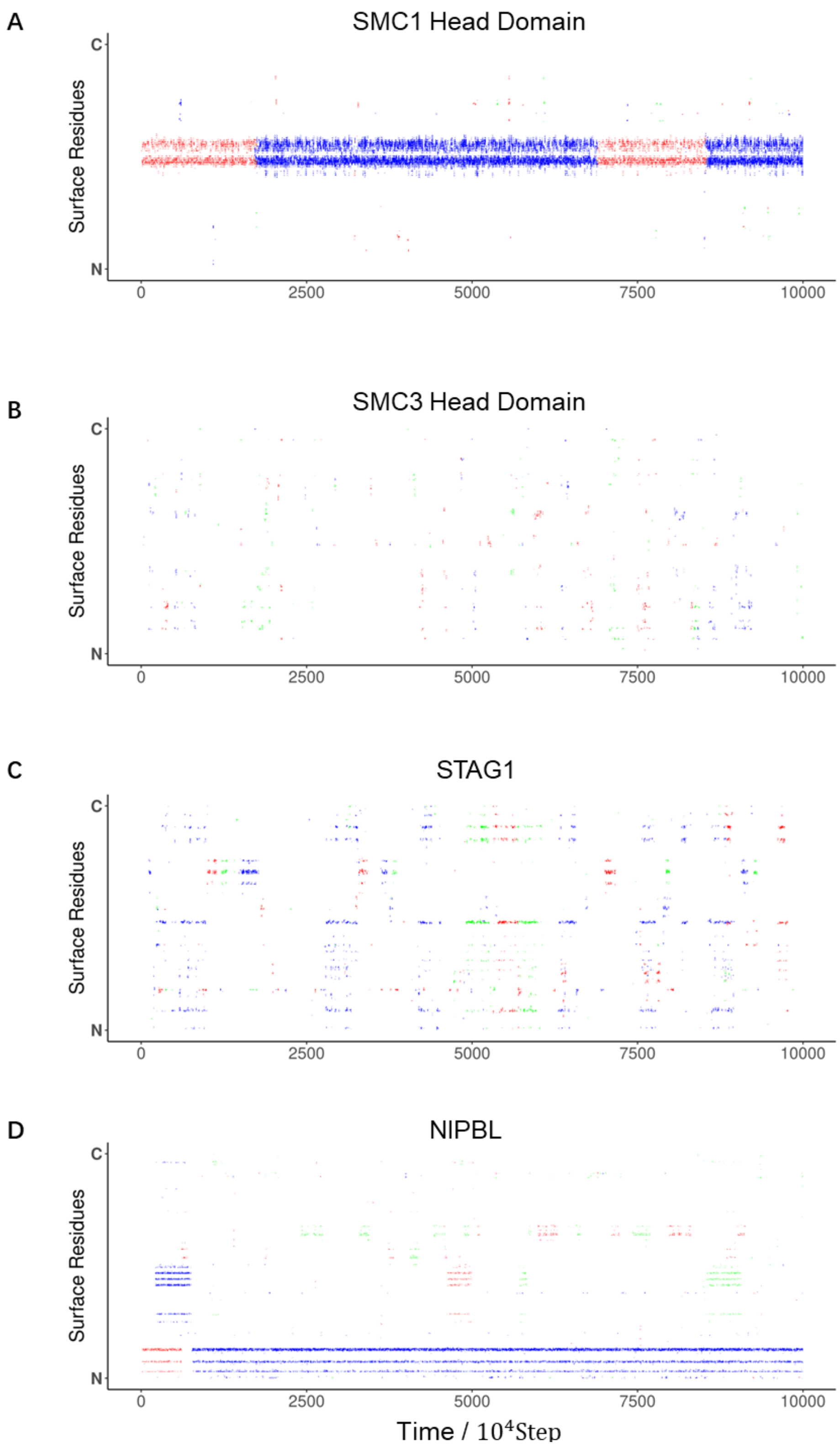


**Fig S6. Example time series of IDs of amino acid particles in contact with DNA.** Visualized in the same way as Fig 3B.
