# Supplementary material for: Molecular dynamics simulations of human cohesin subunits identify DNA binding sites and their potential roles in DNA loop extrusion
Source: PLoS Comput Biol. 2025 Apr 4;21(4):e1012493. doi: 10.1371/journal.pcbi.1012493 (PMC11970657; doi:10.1371/journal.pcbi.1012493)
Supplement: S7 Fig — DNA binding affinity simulation assays were performed using STAG1 structured domain and 20 bp B-type DNA fragment, with CG content of 0.2, 0.5, or 0.8. The dissociation rate constants Koff calculated from assays with different DNA fragments were plotted. (PDF) [file pcbi.1012493.s007.pdf]

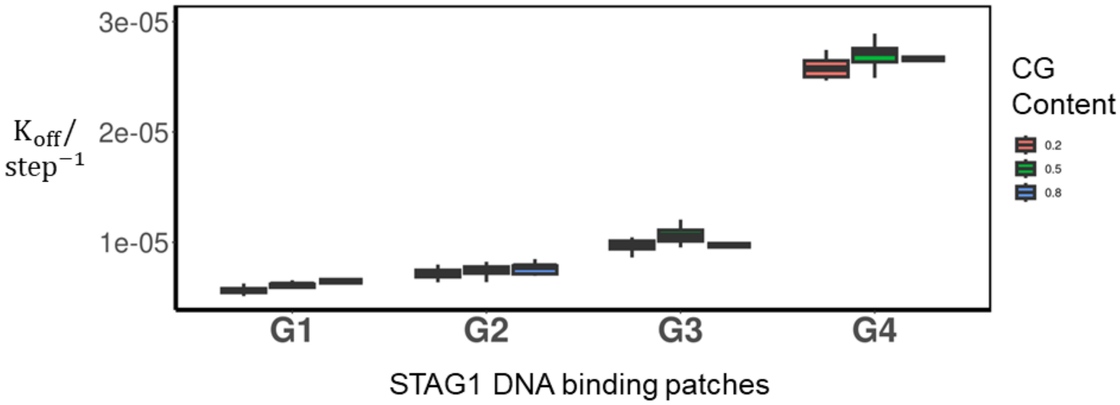

Fig S7. Sequence independent DNA binding affinity. DNA binding affinity simulation assays were performed using STAG1 structured domain and 20 bp B-type DNA fragment, with CG content of 0.2, 0.5, or 0.8. The dissociation rate constants K_{off} calculated from assays with different DNA fragments were plotted.
