# Supplementary material for: Molecular dynamics simulations of human cohesin subunits identify DNA binding sites and their potential roles in DNA loop extrusion
Source: PLoS Comput Biol. 2025 Apr 4;21(4):e1012493. doi: 10.1371/journal.pcbi.1012493 (PMC11970657; doi:10.1371/journal.pcbi.1012493)
Supplement: S8 Fig — (A) Illustration of NIPBL’s “neck” position. (B) The electrostatic potential around the NIPBL HEAT repeats domain calculated and visualized using the APBS plugin [41] in PyMOL [52] (C & D) DNA contact frequency of each amino acid mapped on the coarse-grained model of NIPBL HEAT repeats domain. DNA contact frequency was calculated using simulations conducted with (C) and without (D) RESPAC charge calibration. (PDF) [file pcbi.1012493.s008.pdf]

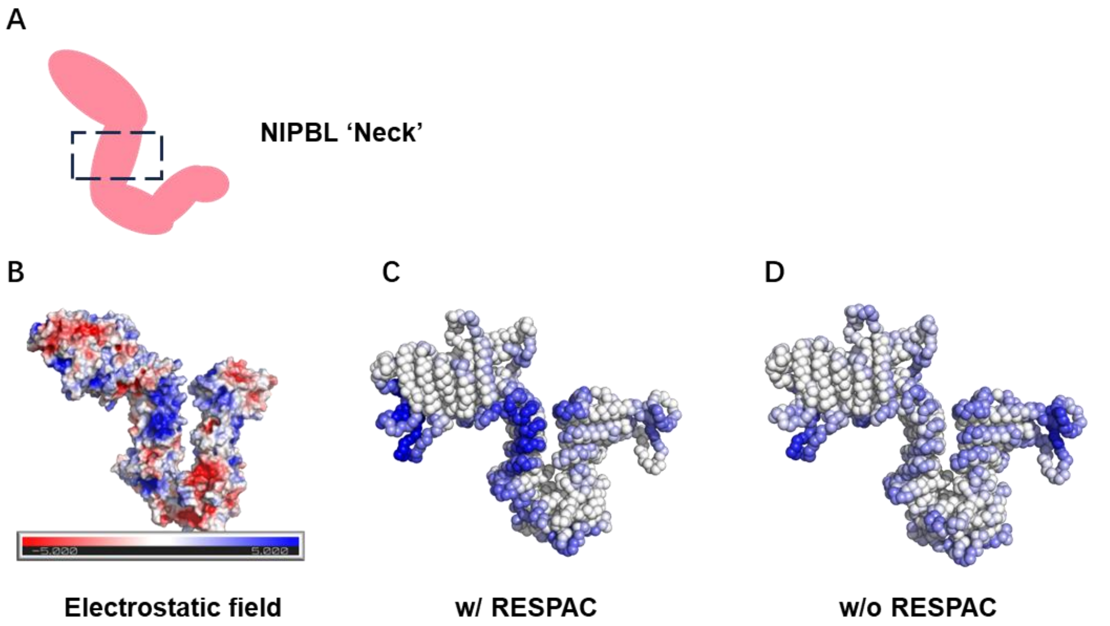

Fig S8. Reproducing surface electrostatic field with RESPAC. (A) Illustration of NIPBL's "neck" position. (B) The electrostatic potential around the NIPBL HEAT repeats domain calculated and visualized using the APBS plugin (1) in PyMOL (2) (C & D) DNA contact frequency of each amino acid mapped on the coarse-grained model of NIPBL HEAT repeats domain. DNA contact frequency was calculated using simulations conducted with (C) and without (D) RESPAC charge calibration.

1. Jurrus E, Engel D, Star K, Monson K, Brandi J, Felberg LE, et al. Improvements to the APBS biomolecular solvation software suite. Protein Science. 2018;27(1):112–28
2. Schrödinger, L. & DeLano, W., 2020. PyMOL, Available at: http://www.pymol.org/pymol
