# Supplementary material for: Molecular dynamics simulations of human cohesin subunits identify DNA binding sites and their potential roles in DNA loop extrusion
Source: PLoS Comput Biol. 2025 Apr 4;21(4):e1012493. doi: 10.1371/journal.pcbi.1012493 (PMC11970657; doi:10.1371/journal.pcbi.1012493)
Supplement: S9 Fig — (A) Strong DNA binding patch A1 (see Table 1, S1 Table) identified by our simulation is colored in red. By aligning the sequence of SMC1’s head domain and emanating coiled-coil arms (observed part in 6WG3) with RAD50’s head domain and emanating coiled-coil arms (observed part in 4W9M) with T-coffee [1], we identified patch A1’s paralogous sequence in RAD50, colored in orange. (B) Molecular docking prediction of SMC1-DNA complex and NIPBL-DNA complex. The strongest DNA binding patches, A1 (SMC1) and H1 (NIPBL), predicted by MD simulations are colored in blue. (C) Molecular docking prediction when specifying positively charged residues in NIPBL’s patch H2 as active sites. The patch H2 predicted by MD simulations is colored in blue. (PDF) [file pcbi.1012493.s009.pdf]

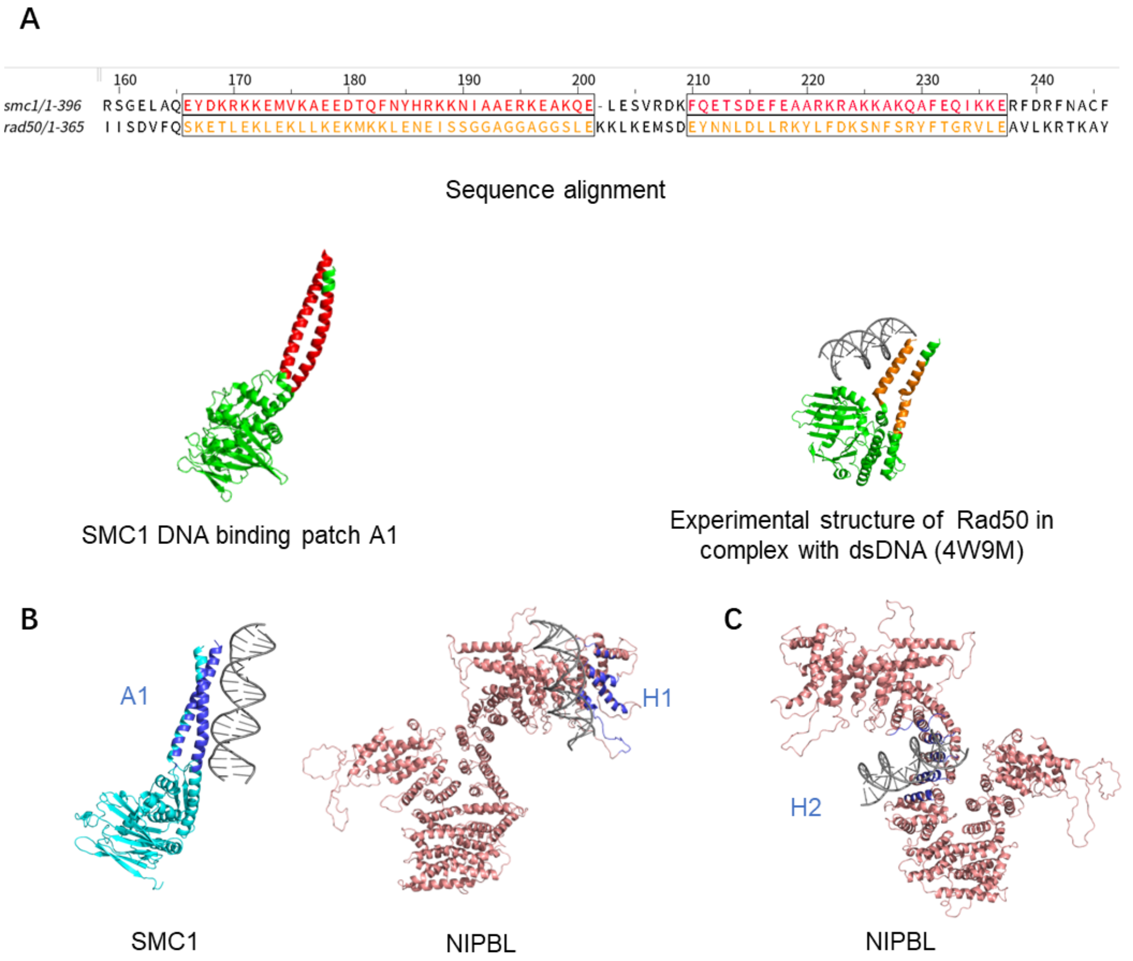

Fig S9. Paralogous protein-DNA complex structures and molecular docking structures as validation for novel predicted DNA binding patches. (A) Strong DNA binding patch A1 (see Table 1, Supplementary Table 1) identified by our simulation is colored in red. By aligning the sequence of SMC1's head domain and emanating coiled-coil arms (observed part in 6WG3) with RAD50's head domain and emanating coiled-coil arms (observed part in 4W9M) with T-coffee (1), we identified patch A1's paralogous sequence in RAD50, colored in orange. (B) Molecular docking prediction of SMC1-DNA complex and NIPBL-DNA complex. The strongest DNA binding patches, A1 (SMC1) and H1 (NIPBL), predicted by MD simulations are colored in blue. (C) Molecular docking prediction when specifying positively charged residues in NIPBL's patch H2 as active sites. The patch H2 predicted by MD simulations is colored in blue.
