# Supplementary material for: Molecular dynamics simulations of human cohesin subunits identify DNA binding sites and their potential roles in DNA loop extrusion
Source: PLoS Comput Biol. 2025 Apr 4;21(4):e1012493. doi: 10.1371/journal.pcbi.1012493 (PMC11970657; doi:10.1371/journal.pcbi.1012493)
Supplement: S10 Fig — (A~B) Steered MD simulation to obtain an SMC1-SMC3 dimer structure with open coiled-coil arms. (C~D) Steered MD simulation to obtain SMC1-SMC3-RAD21 complex. (E~F) Steered MD simulation to obtain the cohesin whole complex structure in which accessory subunits STAG1 and NIPBL are bound only to RAD21 but not SMC1 and SMC3. (F~H) Steered MD simulation and potential switching simulations to obtain cohesin whole complex structure in the ATP bound and DNA bound state in cryo-EM structure 6wg3. (PDF) [file pcbi.1012493.s010.pdf]

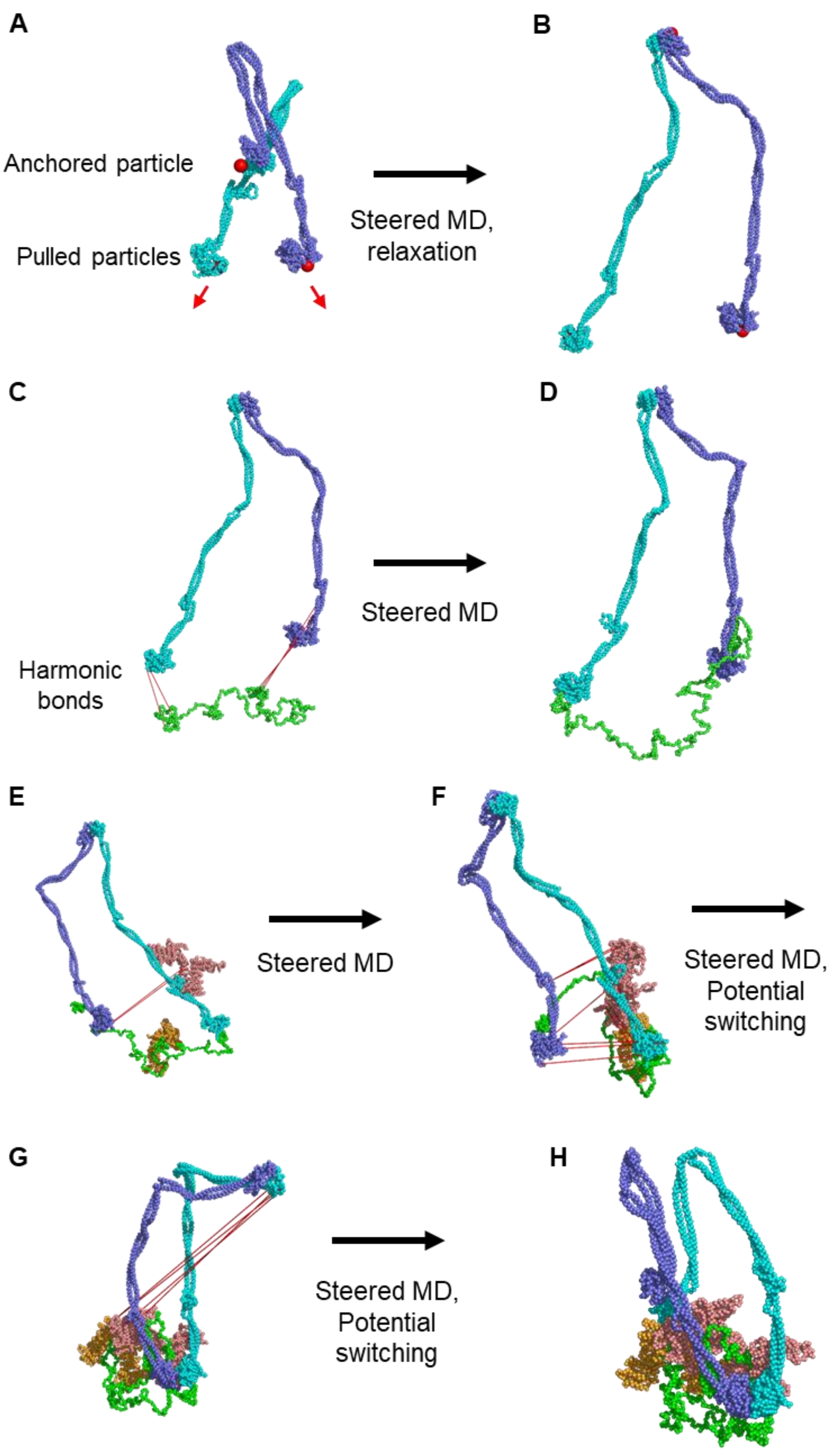

Fig S10. Steered MD setups to construct the whole cohesin complex model. (A~B) Steered MD simulation to obtain an SMC1-SMC3 dimer structure with open coiled-coil arms. (C~D) Steered MD simulation to obtain SMC1-SMC3-RAD21 complex. (E~F) Steered MD simulation

to obtain the cohesin whole complex structure in which accessory subunits STAG1 and NIPBL are bound only to RAD21 but not SMC1 and SMC3. (F~H) Steered MD simulation and potential switching simulations to obtain cohesin whole complex structure in the ATP bound and DNA bound state in cryo-EM structure 6wg3.
