# Supplementary material for: Molecular dynamics simulations of human cohesin subunits identify DNA binding sites and their potential roles in DNA loop extrusion
Source: PLoS Comput Biol. 2025 Apr 4;21(4):e1012493. doi: 10.1371/journal.pcbi.1012493 (PMC11970657; doi:10.1371/journal.pcbi.1012493)
Supplement: S11 Fig — (A) Definition of project plane used in Fig 4D. two vectors x,z define the plane cutting the SMC1-SMC3 ring compartment. x is the vector normal to coiled coil arms emanating from SMC1/3 head dimer, z is the bisector of the SMC1 coiled coil arm and SMC3 coiled coil arm. (B) Convergence analysis of θ. Rolling average of θ in a 5 × 10^5^ step window in all 20 trajectories (black) and mean value of θ in all time steps in all trajectories (red) are plotted. (PDF) [file pcbi.1012493.s011.pdf]

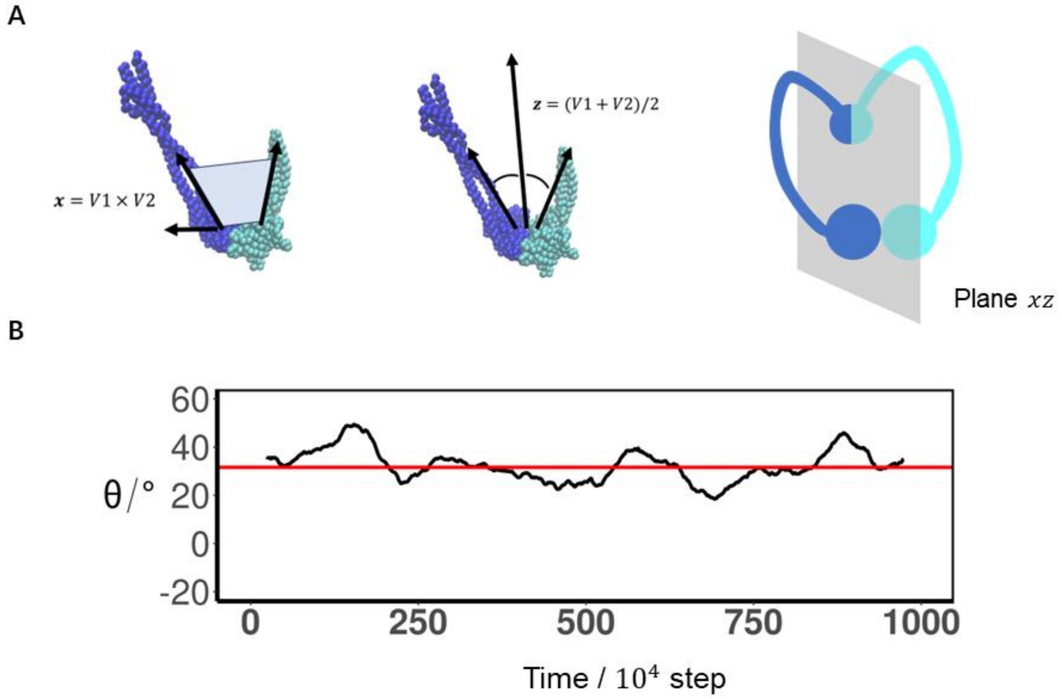

Fig S11. Definition and analysis of DNA bending angle $\boldsymbol{\theta}$. (A) Definition of project plane used in Fig 4D. two vectors $\boldsymbol{x}, \boldsymbol{z}$ define the plane cutting the SMC1-SMC3 ring compartment. $\boldsymbol{x}$ is the vector normal to coiled coil arms emanating from SMC1/3 head dimer, $\boldsymbol{z}$ is the bisector of the SMC1 coiled coil arm and SMC3 coiled coil arm. (B) Convergence analysis of θ. Rolling average of θ in a 5×10^5 step window in all 20 trajectories (black) and mean value of θ in all time steps in all trajectories (red) are plotted.
