# Supplementary material for: Molecular dynamics simulations of human cohesin subunits identify DNA binding sites and their potential roles in DNA loop extrusion
Source: PLoS Comput Biol. 2025 Apr 4;21(4):e1012493. doi: 10.1371/journal.pcbi.1012493 (PMC11970657; doi:10.1371/journal.pcbi.1012493)
Supplement: S12 Fig — (A) The majority of RAD21 IDR is geometrically located between SMC proteins (SMC1 and SMC3) and HEAT repeat proteins (STAG1 and NIPBL). (B) The N-terminus of RAD21’s IDR is on the “back” side of the complex. (C) The C-terminus of RAD21’s IDR is on the “back” side of the complex. (PDF) [file pcbi.1012493.s012.pdf]

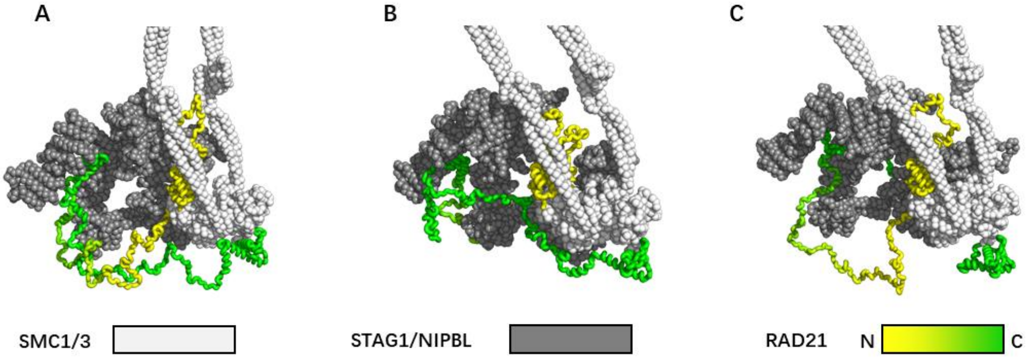

Fig S12. Example cohesin whole complex structures with different RAD21 pathways. (A) The majority of RAD21 IDR is geometrically located between SMC proteins (SMC1 and SMC3) and HEAT repeat proteins (STAG1 and NIPBL). (B) The N-terminus of RAD21's IDR is on the "back" side of the complex. (C) The C-terminus of RAD21's IDR is on the "back" side of the complex.
