# Supplementary material for: Molecular dynamics simulations of human cohesin subunits identify DNA binding sites and their potential roles in DNA loop extrusion
Source: PLoS Comput Biol. 2025 Apr 4;21(4):e1012493. doi: 10.1371/journal.pcbi.1012493 (PMC11970657; doi:10.1371/journal.pcbi.1012493)
Supplement: S13 Fig — Positions of yeast SMC1 (A & B) and SMC3 (C & D) elbow in the crystal structures, compared with the positions of their homolog sequences in human SMC1 and SMC3 in the predicted structures. (PDF) [file pcbi.1012493.s013.pdf]

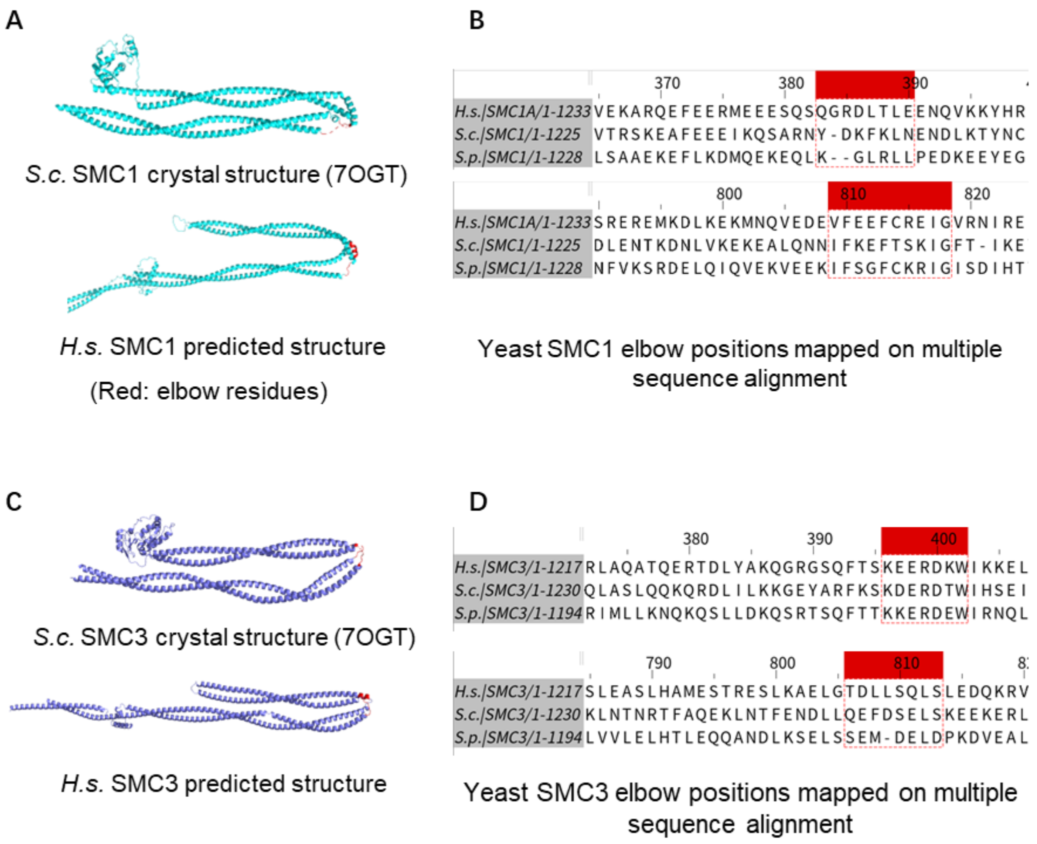

Fig S13. Prediction of SMC1 and SMC3 missing reagions. Positions of yeast SMC1 (A & B) and SMC3 (C & D) elbow in the crystal structures, compared with the positions of their homolog sequences in human SMC1 and SMC3 in the predicted structures.
