# Supplementary material for: Molecular dynamics simulations of human cohesin subunits identify DNA binding sites and their potential roles in DNA loop extrusion
Source: PLoS Comput Biol. 2025 Apr 4;21(4):e1012493. doi: 10.1371/journal.pcbi.1012493 (PMC11970657; doi:10.1371/journal.pcbi.1012493)
Supplement: Attachment — Submitted filename: Response_to_Reviewers.pdf [file pcbi.1012493.s019.pdf]

Here is a point-by-point response to the reviewers' comments and concerns.

Comments from Reviewer 1

Overview: *The manuscript details a considerable body of complex computational work aimed at shedding more light on the structure, function and mechanisms underpinning the activity of cohesin. The approach appears to be a development of the studies on condensin that the team have previously published in PLoS Computational Biology (reference 21). The work is well described, appears to be of high quality, and provides intriguing and experimentally testable ideas about the roles of various potential DNA-binding patches on the complex that contributes to ongoing debate in the area over competing mechanistic hypotheses.*

Response: We would like to thank reviewer 1 for taking the time to read our manuscript and for his/her thoughtful and encouraging comments.

Comment 1: *My only minor reservations relate to a general lack of the application of statistical methods to quantify the precision/significance of observations that are made. So for example, what are the error bars on the dissociation rate constants (and how are they calculated)? What is the evidence that the bending angle investigations (Figure 5) are converged (where do figures like "32.4°±0.3°" on page 8 come from - the data in Figure 5E suggests much greater flexibility and lower if any statistical significance between the behaviour of dimer and tetramer systems).*

Response: We thank the reviewer for pointing out the need for more statistical analysis on the precision and significance of observations. We have now updated the methods used to calculate the dissociation rate constants of each DNA binding patch (Figure 4, Table 1 and Supplementary Table 1) and the DNA bending angle distribution (Figure 5E) and performed the statistical analysis as described in these new sections in the methods:

> "For one binding patch, the lifetime of all binding events involving each DNA fragment in all trajectories was calculated, with the events considered independent. We conducted bootstrapping (30) to estimate the value and error of the dissociation rate constants k_{off}. With N_0 being the total amount of events, the sample size was set the same as N_0, and the samples were drawn 1000 times with replacements. The mean value of k_{off} was used as the strength of the patch, and the standard deviation of k_{off} was used as the error."
>
> "To confirm that the simulation reached convergence, we calculated the rolling average of θ in a moving time window of 500 frames (5×10^5 steps) from all trajectories. The rolling averages showed slight fluctuation but no upward or downward trend over time (Fig S11B). The first half of each trajectory was discarded to avoid bias induced by the initial structure. The mean value of the DNA bending angle $\theta_i|_{i=1,2,\ldots,20}$ was calculated for each trajectory. θ_i calculated from each trajectory was considered independent. The mean value of θ_i was used

as the DNA bending angle θ in the result section. In the case of the SMC1/3 head dimer and 72 bp DNA simulation system, only the 4 trajectories in which DNA did not dissociate from protein domain throughout the simulation were used to calculate θ_i."

Comment 2: *More qualitatively, it would be good to have some commentary on how deterministic the large-scale model building work has been. Both MODELLER and steered MD simulation methods can potentially generate significantly different predictions (maybe) from run to run - what sort of variability was observed here - what structural features can be regarded as "well resolved" and which should be regarded as more approximate?*

Response: We agree with this suggestion and have added information on the determinism of large-scale model building, clarifying:

"We used MODELLER to generate 5 structures for each protein or protein domain and selected the structure with the best score. MODELLER consistently predicted the position of secondary structures and loops."

When building the whole cohesin complex model in a DNA gripping state using steered MD, the heterogeneity mainly comes from the different possible pathways RAD21's IDR takes from its N-terminus near the SMC3 head to its C-terminus near the SMC1 head. We modeled a few different possible structures and showed them in Figure S12. On the other hand, structures of SMC1, SMC3, STAG1 and NIPBL in the gripping state can be considered well resolved.

<u>Comments from Reviewer 2</u>

Overview: *Gu et al. present a molecular dynamics study of human cohesion subunits and identify DNA binding sites, suggesting functional roles for these in DNA loop extrusion. The study involves the use of reduced representation molecular dynamics simulations (including steered MD) to simulate DNA-protein interactions for a variety of cohesion models (single subunits and dimers), and identifies proposed binding sites for DNA.*

Response: We sincerely appreciate reviewer 2's recognition of our work and his/her acknowledgment of the methods and findings we presented.

Comment 1: *An overarching comment I would make is that whilst the simulations are employed appropriately, a single method is used throughout the study, and whilst the authors suggest potential mutations that could be validated experimentally, they could also do some level of validation themselves using other techniques such as all-atom molecular dynamics (AT-MD) or docking/binding calculations using eg. FoldX based on frames from their simulations.*

Response: We thank the reviewer for highlighting the lack of multiple methods to support our

findings. To address this concern, we have employed the docking tool HADDOCK to predict DNA binding sites on cohesin subunit protein domains, which provides an independent validation of our approach. The following Methods and Results section has been added:

> "We used the all-atom resolution structures of SMC1 head domain or structured regions of NIPBL as input structure 1 in the HADDOCK 2.4 web server (30), and the all-atom resolution structure of 20 bp B-type DNA ATAGTGATTATGAAAACTTT as input structure 2.
>
> All surface residues in the protein structure were set as "passive", and all residues in the DNA were set as "active" so that HADDOCK searches for the docking interface on the entire surface. In each HADDOCK run, the positively charged residues in one of the patches were set as "active" to validate each specific DNA binding patch predicted by our MD simulation."
>
> "We used the molecular docking tool HADDOCK (30) to validate our predicted DNA binding patches independently. We used one protein domain (SMC1 head domain [A] or NIPBL HEAT repeats domain[H]) and one 20 bp DNA fragment as input structures. Without specifying the interface regions, the DNA-protein docking interfaces predicted by HADDOCK overlap with the strongest DNA binding patches of each protein domain predicted by our MD simulations, A1 and H1 (Table 1, Fig S9B). A1 and H1 were novel DNA binding patches not studied in previous mutagenesis studies. When specifying positively charged surface residues selected from our other predicted patches as interface regions in HADDOCK input parameters, HADDOCK produced complex structures in which DNA docks to the corresponding patch (Fig S9C)."

As for the potential mutations we suggested, we hypothesize that DNA binding patches H1 on NIPBL and A1 on SMC1/3 head dimer can participate in activities proposed in DNA loop extrusion models like the swing and clamp model. If mutating one of these sites abolishes loop extrusion, this can be used as evidence that these patches are involved in loop extrusion, increasing the credibility of the corresponding loop extrusion model. However, since said activities occur in steps other than the DNA gripping step, our current model cannot study the impact of mutation on patches H1 and C1. Therefore, we did not include validations using in-silico mutagenesis methods.

Comment 2: *Standard of English could use some slight improvement. The manuscript is fully readable in current state, but grammar is not fully correct. I would recommend having the manuscript proofed.*

Response: We would like to thank reviewer 2 for his/her feedback. We have carefully reviewed and revised the manuscript to improve its English grammar and overall readability.

Comment 3: *Is the DNA sequence important for binding? The authors have only simulated one*

sequence (reasonably – as simulating many would exponentially increase the simulation time required), is this likely to be meaningful? If there is a specific sequence/CG rich region that binds, and if this happens to be eg. On the end of DNA sequence, will this not bias the simulations somewhat?

Response: We acknowledge that the choice of a specific DNA sequence could potentially introduce a bias in the results. Cohesin is commonly considered a non-sequence-specific DNA binding protein complex because of its ability to establish loop structures across the whole genome, but a bias may exist. To address this concern, we performed additional DNA binding simulations of the STAG1 structured domain with various DNA sequences differing in CG content (20%, 50%, and 80%), shown in Figure S7. The results indicated that the sequence dependence of the binding affinity is minimal.

Comment 4: *Have the authors considering performing preliminary validation themselves? I could imagine short scale all atomistic (AT) MD simulations, or energy calculations such as FoldX could be used to induce proposed mutations and calculate DNA binding affinity differences? I would expect some sort of validation of this type (eg. Multiscale MD) to be performed for publication in this journal.*

Response: As mentioned in our response to comment 1, because our current model only includes the DNA gripping state, inducing mutation in all-atom MD simulations or energy calculations alone cannot validate our hypotheses, as models of other steps in loop extrusion are required to study the impact of the induced mutations. Therefore, we do not include mutagenesis in this manuscript. However, as explained above, we have validated the docking site using HADDOCK.

Comment 5: *Leading on from the previous comment – the authors suggest amino acids found to be critical experimentally and that overlap with their binding patches. Are there other critical residues that are not overlapping? I.e. have the authors found that a majority of the critical residues are in their patches, or have they cherry picked a subset that happen to fall into their binding sites? Statistics could help here, or simulations/small scale docking studies of some of the mutations to show they are consistent.*

Response: We would like to thank reviewer 2 for pointing out the ambiguity of significance in our statement. We have updated the manuscript, clarifying that there are 9 mutation/deletion sites studied by Bauer *et al.* (reference 9), and all 9 overlapped with our predicted patches. Therefore, there are no critical experimental residues not found among the patches identified from the simulation, providing a strong validation of our results.

Comment 6: *Statistics could be performed to prove the difference between DNA bending angles*

in the different systems (Figure 5E). The difference looks significant to me but I would suggest calculating it and presenting it to bolster your claim that it is different.

Response: We acknowledge this point addressed in the revised manuscript, as described above in response to comment 1 from reviewer 1.

Comment 7: *The remarks under Table 1 are not numbered in the document I received (may be a rendering issue in the journal format).*

Response: We apologize for the inconvenience caused by the previous rendering issues. We replaced the automatic numbering with plain text, which should prevent such issues.

<u>Comments from Reviewer 3</u>

Overview: *The submitted manuscript studies the mechanism of DNA loop extrusion by the SMC (structural maintenance of chromosomes) complex,particularly focusing on cohesion. Using molecular dynamics simulations with AICG2+ and 3DNA models , the authors look to identigy DNA-binding sites across cohesin subunits (SMC1, SMC3, STAG1, NIPBL) and assess their role in facilitating DNA loop extrusion. DNA-binding patches were mapped to the proteins, their binding affinities were computed and finally models were constructed to explain how these interactions might drive the loop extrusion process. The study provides insights into possible ways of DNA handover during loop extrusion and highlights the cooperative roles of accessory subunits such as STAG1 and NIPBL.*

Response: We would like to thank reviewer 3 for his/her detailed review and thoughtful summary of our work.

Comment 1: *The manuscript is well presented although in some cases it could benefit of a bit more rigorous details: For instance, how many initial modeller structures were used? Does the initial shape of the modelled loop affect your simulations? Please do comment on that. Perhaps it would be useful to comment how the induced fitting is modelled with the proposed methodology for the untrained reader.*

Response: We agree with this suggestion and have added information on the mentioned details. We modeled 5 structures of each subunit using MODELLER, and this software produced highly consistent structures. We chose the structure with the best MODELLER score for each subunit model and only used one structure of each subunit when modelling the whole cohesin complex in the DNA gripping state. The initial placement of subunits in the steered-MD step affects the pathway RAD21's IDR takes from its N-terminus near the SMC3 head to its C-terminus near the SMC1 head. We modeled a few different possible structures and showed them in Figure S12. On

the other hand, structures of SMC1, SMC3, STAG1, and NIPBL in the gripping state can be considered deterministic. This point has also been addressed in our response to comment 2 from reviewer 1.

Comment 2: *A point of concern to me is, if the choice of modelling only 1 random sequence in the small DNA duplexes binding essay affect the assignation of binding patches in the protein. Perhaps several randomly generated sequences would be useful to avoid any bias and increase statistical significance. Are they replicas or just one single simulation run? This should be addressed. How does this model compare to traditional flexible docking approaches to recognise the binding sites?*
Response: We acknowledge the reviewer's concerns about potential sequence bias and comparison against docking predictions. We addressed them in the revised manuscript by performing new simulations with different DNA sequences and docking with HADDOCK, as described in our responses to comments 3 and 1 from reviewer 2, respectively.

Comment 3: *The remarks labels in Table 1 are not shown (maybe got lost after pdf creation?)*
Response: We apologize for the inconvenience caused by the previous rendering issues. We replaced the automatic numbering with plain text, as mentioned in the response to comment 7 from reviewer 2.

Final remark: *Overall, the manuscript presents a reasonable mechanism for DNA loop extrusion mediated by cohesins, using a simulation approach. The findings offer valuable predictions for future experimental research and expand the knowledge on how cohesins may interact with DNA during loop extrusion. It could use some improvements in clarity and discussions on experimental implications – and lack thereof- This study has the potential to be a significant addition to the field.*
Response: Again, we would like to thank reviewer 3 for his/her thoughtful feedback and for highlighting both the strengths of our work and areas for improvement.

Additional clarification:
All data and code underlying the findings described in the manuscript is deposited at The Biological Structure Model Archive. (https://bsma.pdbj.org/). The data entry will be public after the manuscript is published. Meanwhile, we can provide a temporary link to the reviewers and editors to review the deposited data upon request.
