# Supplementary material for: Molecular dynamics simulations of human cohesin subunits identify DNA binding sites and their potential roles in DNA loop extrusion
Source: PLoS Comput Biol. 2025 Apr 4;21(4):e1012493. doi: 10.1371/journal.pcbi.1012493 (PMC11970657; doi:10.1371/journal.pcbi.1012493)
Supplement: Attachment — Submitted filename: Response_to_Reviewers_auresp_2.pdf [file pcbi.1012493.s020.pdf]

Dear Editor,

We would like to thank the editor for giving us the opportunity to submit a revised draft of our manuscript titled "Molecular dynamics simulations of human cohesin subunits identify DNA binding sites and their potential roles in DNA loop extrusion" to PLOS Computational Biology. We appreciate the time and effort the editor and the reviewers dedicated to providing valuable feedback on our manuscript. We have been able to incorporate changes to reflect the suggestions provided by the reviewers. We have highlighted the changes in the revised manuscript in red text.

Here is a point-by-point response to the reviewers' comments and concerns.

Comments from Reviewer 1

Overview: *The authors appear to have responded very positively to the comments I made in my original review, I have no further concerns.*

Response: We would like to thank reviewer 1 for recognizing the improvements we have made.

Comments from Reviewer 2

Overview: *The authors have made a good faith attempt to address comments from all reviewers, and have included significantly more analysis and work to address my and the other reviewers comments. In particular the inclusion of further docking to validate findings from the simulations, and addition of statistics has significantly improved the validity of the manuscript.*

Response: We would like to thank reviewer 2 for recognizing the improvements we have made, and for their thoughtful summary of our additional contents in the revised manuscript.

Comments from Reviewer 3

Overview: *Many aspects have been clarified in this version, however I still have some comments:*

Response: We would like to thank reviewer 3 for recognizing the improvements we have made and thank them for their comments on the remaining issues. We would like to address them here.

Comment 1: *When mentioning the choice of the best models from MODELLER, the name of the scoring method is missing.*

Response: We added information on the scoring method in Materials and methods section:

"We used MODELLER to generate 5 structures for each protein or protein domain, selected the model with the lowest value of the MODELLER objective function (molpdf) as the best model.

The MODELLER consistently predicted the position of secondary structures and loops."

Comment 2: *Then new docking assay with HADDOCK was done with the structures of SMC1 head domain, whereas the new binding assays with different GC content were done on the STAG1 subunit. What is the rationale behind the choice of different cohesin subunits?*

Response: We performed HADDOCK on cohesin subunit monomer systems SMC1 Head domain, SMC3 Head domain, STAG1, and NIPBL. For each system, the protein-DNA docking interfaces predicted by HADDOCK overlap with the strongest DNA binding patch predicted by our MD simulations. We showed the figures of SMC1 head and NIPBL head domain docking results because these two results validate two novel DNA binding patches, A1 and H1, predicted in this study. The docking results of other proteins were not included due to space limitations. We did not choose SMC1 head for the new binding assay studying DNA sequence-specificity because factors other than DNA sequence also affect the calculated DNA binding affinity of DNA binding patch A1, as described in the section "Molecular dynamics simulations predicted DNA binding patches in cohesin":

"… we observed strong patches A1 and C1 (Table 1), corresponding to the same group of amino acids on the coiled-coil part connected to the SMC1 head domain. These patches were always bound to at least one DNA segment throughout the simulation trajectories. This means the dissociation of one DNA segment from these patches resulted from another DNA segment competing for the same binding site (Fig S6). Although association duration for these patches still follows exponential distributions, the dissociation rate constants calculated under this condition have different physical meanings than other patches and should not be compared directly."

Instead, we chose STAG1 as the target system because it contains multiple DNA binding patches with a wide range of dissociation rate constants.

Comment 3: *Regarding the bias on DNA sequence, the authors should report the new DNA sequences with different GC content that were used too. Is there a reason for choosing to only run it for STAG1 and not all the other subunits? This should be clarified - ideally, it would be good to have it for all subunits for completeness.*

Response: We added reports on the DNA sequence used in the "Materials and Methods" section: "A series of simulations studying the effect of different DNA sequences on affinity is performed with STAG1 and 3 B-type DNA fragments with the same sequence. In different simulation trajectories, the DNA sequence is either TCAGGTTTCACAGGGACAAA (45% GC content) or

GGCCCCAAGCGCGGGTTCCC (80% GC content)."

Considering the cost of time and computational resources, we did not perform assays regarding the bias induced by DNA sequence on all cohesin subunits. The rationale for only choosing STAG1 was clarified in the section "Molecular dynamics simulations predicted DNA binding patches in cohesin":

"STAG1 contains multiple DNA binding patches, including both strong patches (dissociation rate constant below 2.0×10^{-5}) and weak patches (dissociation rate constant above 2.0×10^{-5}), and does not contain the patch that cannot be precisely quantified (patch A1, described above"). We consider the DNA binding patches on STAG1 a good sample of all the DNA binding patches identified in this study to eliminate the possibility of DNA sequence-induced bias."

Overall, we reasoned this would be the optimal representative system to evaluate a potential DNA sequence dependence on binding affinity, which we showed to be very weak.

Additional clarification:

1) The "Fig 9F to H" mentioned in the text on page 16 is a typo of "Fig S10F to H." The error has been corrected.

2) All data and code underlying the findings described in the manuscript were deposited at the Biological Structure Model Archive at the link https://bsma.pdbj.org/entry/70, which is publicly available now.
